# Pre-trial inter-laboratory analytical validation of the FOCUS4 personalised therapy trial

**DOI:** 10.1136/jclinpath-2015-203097

**Published:** 2015-09-08

**Authors:** Susan D Richman, Richard Adams, Phil Quirke, Rachel Butler, Gemma Hemmings, Phil Chambers, Helen Roberts, Michelle D James, Sue Wozniak, Riya Bathia, Cheryl Pugh, Timothy Maughan, Bharat Jasani

**Affiliations:** 1 Department of Pathology and Tumour Biology, Leeds Institute of Cancer and Pathology, St James Hospital, Leeds, UK; 2 Institute of Cancer & Genetics, Cardiff University School of Medicine, Velindre Hospital, Cardiff, UK; 3 Cardiff and Vale UHB-Medical Genetics University Hospital of Wales, Heath Park, Cardiff, UK; 4 Cardiff and Vale UHB- Histopathology University Hospital of Wales, Heath Park, Cardiff, UK; 5 MRC Clinical Trials Unit at UCL, London, UK; 6 Gray Laboratories, CRUK/MRC Oxford Institute for Radiation Oncology, University of Oxford, Oxford, UK; 7 Institute of Cancer and Genetics, Heath Park, Cardiff, UK

**Keywords:** COLORECTAL CANCER, ANTIBODIES, LABORATORY TESTS, MOLECULAR PATHOLOGY

## Abstract

**Introduction:**

Molecular characterisation of tumours is increasing personalisation of cancer therapy, tailored to an individual and their cancer. FOCUS4 is a molecularly stratified clinical trial for patients with advanced colorectal cancer. During an initial 16-week period of standard first-line chemotherapy, tumour tissue will undergo several molecular assays, with the results used for cohort allocation, then randomisation. Laboratories in Leeds and Cardiff will perform the molecular testing. The results of a rigorous pre-trial inter-laboratory analytical validation are presented and discussed.

**Methods:**

Wales Cancer Bank supplied FFPE tumour blocks from 97 mCRC patients with consent for use in further research. Both laboratories processed each sample according to an agreed definitive FOCUS4 laboratory protocol, reporting results directly to the MRC Trial Management Group for independent cross-referencing.

**Results:**

Pyrosequencing analysis of mutation status at *KRAS* codons12/13/61/146, *NRAS* codons12/13/61, *BRAF* codon600 and *PIK3CA* codons542/545/546/1047, generated highly concordant results. Two samples gave discrepant results; in one a *PIK3CA* mutation was detected only in Leeds, and in the other, a *PIK3CA* mutation was only detected in Cardiff. pTEN and mismatch repair (MMR) protein expression was assessed by immunohistochemistry (IHC) resulting in 6/97 discordant results for pTEN and 5/388 for MMR, resolved upon joint review. Tumour heterogeneity was likely responsible for pyrosequencing discrepancies. The presence of signet-ring cells, necrosis, mucin, edge-effects and over-counterstaining influenced IHC discrepancies.

**Conclusions:**

Pre-trial assay analytical validation is essential to ensure appropriate selection of patients for targeted therapies. This is feasible for both mutation testing and immunohistochemical assays and must be built into the workup of such trials.

**Trial registration number:**

ISRCTN90061564.

## Introduction

**Figure 1:**
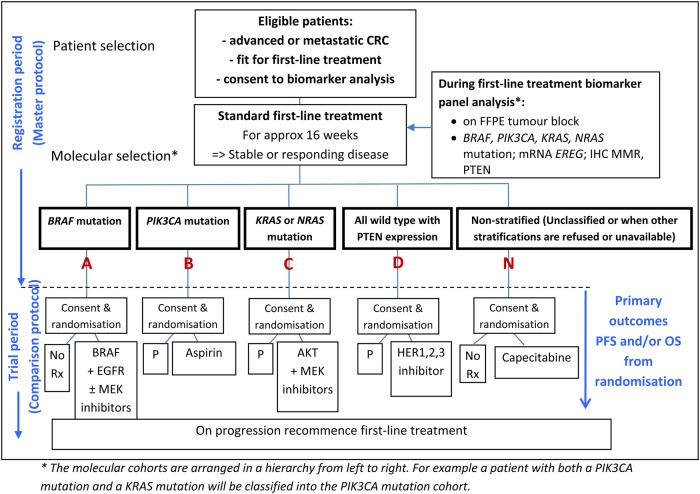
Schematic representation of FOCUS4. *Hierarchical ordering of the molecular cohorts from A through N. CRC, colorectal cancer; EREG, epiregulin; EGFR, epidermal growth factor receptor; FFPE, formalin-fixed, paraffin-embedded; HER, human epidermal growth factor receptor; IHC, immunohistochemistry; MMR, mismatch repair, OS, overall survival; P, placebo; PFS, progression-free survival; Rx, treatment. http://www.focus4trial.org/.

Molecular characterisation of tumours is leading to increasing personalisation of cancer therapy tailored to an individual and the target cancer. FOCUS4 (figure 1) marks a significant advancement in terms of clinical trial design.^1^ It aims to stratify patients to novel targeted agents by a prospective, progressive molecular stratification process. Following patient registration, archival formalin-fixed, paraffin-embedded (FFPE) tumour samples from patients with advanced or metastatic colorectal cancer (CRC) will be sent from collaborating centres to undergo mutation and immunohistochemical testing at one of the two centralised testing laboratories in Leeds and Cardiff, to allow the allocation of patients into molecular cohorts, within which there is a specific randomised comparison.

The current panel of molecular markers selected for the trial is based on biomarkers which have been identified or are hypothesised as having the capacity to predict responses to specific targeted therapies. The trial is designed to allow the panel to progressively change during the life span of the trial, with identification of novel biomarkers and new treatment approaches being incorporated for differing biomarker-defined cohorts.^1^

Mutational analysis of *KRAS* and *NRAS* is already used routinely to determine suitability to receive anti-epidermal growth factor receptor (EGFR) therapy.^2–5^ Mutational status of *BRAF* is becoming widely recognised as a prognostic marker, with the presence of an activating mutation in stage IV being associated with very poor prognosis.^2^ ^6–8^ Mutational activation of *PIK3CA*^9^ or loss of pTEN protein expression^10^ ^11^ has been implicated in driving signalling through the AKT pathway, which is a feature in up to 30% of CRC. While not being used initially to randomise patients, mismatch repair (MMR) status is being determined in order to further stratify patients in the post-randomisation phase.

The aim of this study was to carry out a pre-trial analytical validation between the two designated biomarker testing laboratories in Leeds and Cardiff, in order to identify the inter-observer laboratory agreement on 97 samples of stage IV CRC and to ensure that laboratory testing was accurate and fit for purpose in both laboratories.

## Materials and methods

### Samples

Ninety-seven FFPE tumour resection blocks, from patients previously entered into either the FOCUS3 trial^12^ or consented outside clinical trial to the Wales Cancer Bank (WCB), and stored with prior consent for further use in research, were retrieved from the WCB. The matched diagnostic biopsy blocks were also retrieved in 14 cases, to reflect the fact that 20–40% of patients in FOCUS4 will only have biopsy samples available for analysis. From the outset, these biopsies were only intended to be used for pTEN immunohistochemical analysis. For the purpose of this validation, the blocks were anonymised before sending to the biomarker teams in the laboratories in Cardiff and Leeds. All blocks were initially sent to the Cardiff laboratory for processing, before being forwarded to Leeds for identical processing. Both laboratories were therefore able to carry out all assay analyses completely independently, representative of the process of analysis from sample receipt, to the reporting of the results to the Clinical Trials Unit.

### Sample processing

A series of 5 µm thick sections were taken from each block, the first of which was used for H&E staining, to identify the area of greatest tumour density, and the rest made available for DNA extraction and whole section (w/s) immunohistochemistry (IHC). From the residual blocks, tissue microarrays (TMAs) were then created comprising four 0.6 mm tumour tissue cores and one core, if available, of ‘tumour-associated’ normal tissue. In order to reduce tissue use, the TMAs were only prepared once, in Cardiff, and then shipped to Leeds, where sections were cut and used for IHC.

### DNA macrodissection and extraction

The spare sections from the resection blocks were marked out for the richest areas of neoplastic cell content, using the corresponding H&E-stained section as a guide, and macrodissected. DNA was extracted in Leeds using the QIAGEN QIAamp DNA Extraction Kit (QIAGEN, Skelton House, Manchester, England, UK), and in Cardiff using the manufacturer's standard protocol, on the QIAGEN EZ1 (QIAGEN, Skelton House, Manchester, England, UK).

### Mutation detection

Analysis of mutation hotspots within *KRAS* codons 12, 13, 61 and 146 (exons 2, 3 and 4), *BRAF* codon 600 (exon 15), *NRAS* codons 12, 13 and 61 (exons 2 and 3) and *PIK3CA* codons 542, 545, 546 and 1047 (exons 9 and 20) was carried out by pyrosequencing. Pyrosequencing was carried out in each lab using a PyroMark Q96 (QIAGEN, Skelton House, Manchester, England, UK) (see online supplementary appendix 1). A negative water control and a positive control for each assay were included in every sample run. Raw data files were used to generate pyrograms for interpretation by qualified personnel.

### MMR status determination

**Figure 2:**
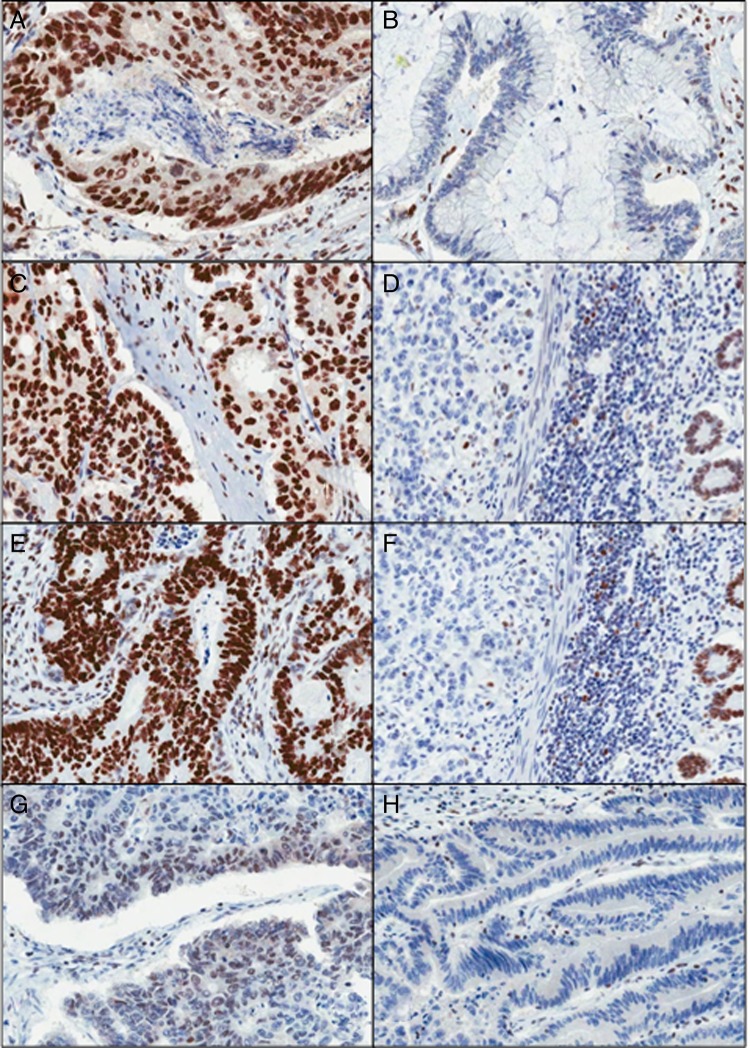
Mismatch repair immunohistochemistry (MMR IHC). (A and B) Positive and negative MLH1 tumours are shown, respectively. (C and D) Positive and negative MSH2 tumours are shown, respectively. (E and F) Positive and negative MSH6 tumours are shown, respectively. (G and H) Positive and negative PMS2 tumours are shown, respectively (×200 magnification).

All four immunohistochemical analyses were carried out on a DAKO Autostainer Link 48 (DAKO, Ely, England, UK) using DAKO pre-programmed protocols, available with the Autostainer. Antigen retrieval was performed in the accompanying PT-Link chamber with High pH DAKO Target Retrieval Solution, according to manufacturer's instructions (DAKO, Ely, England, UK). Slides were rinsed with DAKO wash buffer prior to loading into the Autostainer. DAKO ready-to-use antibodies were used for MLH1 (IR079), MSH2 (IR085) and MSH6 (IR086). DAKO PMS2 (M3674) was used at a dilution of 1:40. Sections from the two validation TMAs were stained with each Ab, then corresponding whole sections were also stained in cases where the cores appeared negative or equivocal or for cases where all cores had been lost from the TMA section. Tumours were deemed positive, if any proportion of the tumour nuclei was positively stained, or negative, where all discernible tumour nuclei were negative, in the local presence of positively staining stromal and infiltrating lymphocytic cells (figure 2). Any samples appearing wholly negative with respect to both tumour and stromal components were deemed to be of indeterminate status.

### pTEN protein expression

**Figure 3:**
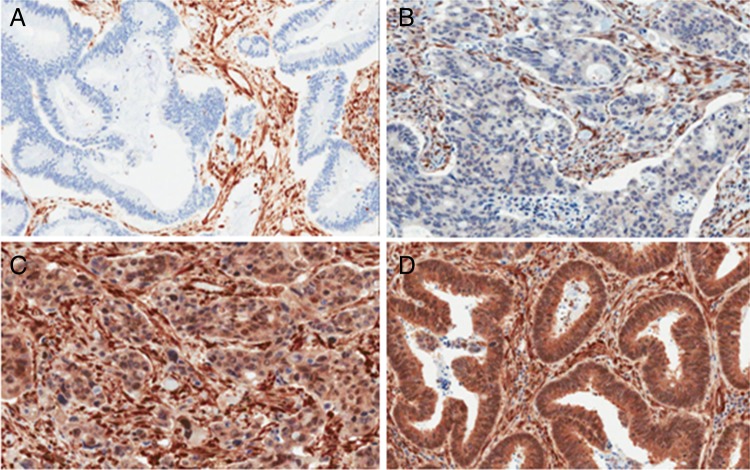
pTEN protein expression. (A) Negative, (B) grade 1—weak cytoplasmic staining, less intense than the surrounding stroma, (C) grade 2—moderate cytoplasmic staining, where staining is equal in intensity to the adjacent stromal staining and (D) grade 3—strong cytoplasmic staining, where staining is stronger in intensity to the adjacent stromal staining (×200 magnification).

Immunohistochemical staining was carried out using the DAKO Autostainer Link 48 (DAKO, Ely, England, UK). Antigen retrieval was carried out in the accompanying PT-Link chamber with High pH DAKO Target Retrieval Solution, according to manufacturer's instructions. Slides were rinsed with DAKO wash buffer prior to loading into the Autostainer. DAKO pTEN Ab (M3627) (DAKO, Ely, England, UK) was used at a pre-determined dilution of 1:100. Both validation TMAs were stained, along with all corresponding whole sections. The presence and intensity grade of cytoplasmic staining in the tumour component was noted (0=negative; 1=weak cytoplasmic staining, less intense than the surrounding stroma; 2=moderate cytoplasmic staining, where staining is equal in intensity to the adjacent stromal staining and 3=strong cytoplasmic staining, where staining is stronger in intensity to the adjacent stromal staining) (figure 3). For the purposes of randomised stratification of patients, any positive result was reported as ‘no loss’ of pTEN; whereas the negative result was reported as ‘absence’ of pTEN. Three FFPE cell lines (LNCaP, pTEN negative; ZR-75-1, a weak expresser of pTEN and MCF7 which overexpresses pTEN) were used to create a mini control TMA, which was stained along with each section. A suspension was generated from each cell line, which was subsequently spun down, fixed in 10% neutral-buffered formalin (NBF), added to 12% Noble agar at a 1:1 ratio, processed and paraffin embedded. Three cores were taken from each and embedded into a new paraffin block to create the mini ‘control TMA’. A section of this was cut onto the same slide as each of the 97 validation samples.

### Data validation

Each laboratory sent their results of all analyses directly to the Medical Research Council (MRC) Clinical Trials Unit for independent cross-referencing. Any discrepant results were discussed between the biomarker teams from both laboratories until a final unanimous result was agreed upon.

## Results

### Mutation detection

The 97 resection samples were subjected to mutation detection by pyrosequencing at the following mutation hotspots: *KRAS* 12/13, *KRAS* 61, *KRAS* 146, *BRAF* codon 600, *NRAS* 12/13, *NRAS* 61, *PIK3CA* exon 9 and *PIK3CA* exon 20. Mutation rates at each mutation hotspot were as expected (table 1). Two samples in Leeds and three samples in Cardiff were deemed to have ‘failed’ as they only passed ≤3 assays. There were only two discrepant cases between the two laboratories (table 2). The first of these was deemed *PIK3CA* wild type (WT) in Cardiff, but shown to contain an exon 9 mutation (c.1633G>A) in Leeds. The second sample was *PIK3CA* WT in Leeds, but found to contain an exon 20 mutation (c.3140A>G) in Cardiff.

**Table 1:** The percentage of mutations found at each mutation hotspot shown for the labs in Leeds and Cardiff

Assay	Leeds (% mutations)	Cardiff (% mutations)
*KRAS* codons 12/13	32/97 (33)	31*/94 (33)
*KRAS* codon 61	3/96 (3.1)	3/95 (3.2)
*KRAS* codon 146	1/95 (1.1)	1/92 (1.1)
*BRAF* codon 600	12/96 (12.5)	12/94 (12.8)
*NRAS* codons 12/13	2/95 (2.1)	2/93 (2.1)
*NRAS* codon 61	2/95 (2.1)	2/95 (2.1)
*PIK3CA* codons 542–546	10/95 (10.5)	9/94 (9.6)
*PIK3CA* codon 1047	1/96 (1.0)	2/93 (2.1)

The percentages reflect the number of samples which yielded a result.

*The discrepancy in mutation detection at *KRAS* codons 12/13 is due to the fact that one of the samples which failed testing in Cardiff was found to have a mutation when tested successfully in Leeds.

**Table 2:** Summary of failed and discrepant cases between laboratories

	Leeds	Cardiff
Failed* samples	14001 and V058	24002, L366 and V058
Partial fails†	None	None
Discrepant samples		
(*PIK3CA* exon 9)	R225 (c.1633G>A)	R225 (WT)
(*PIK3CA* exon 20)	L722 (WT)	L722 (c.3140A>G)

*A failed sample was classed as a sample where ≤3 assays were amplified successfully.

†A partial fail was classed as a sample where not every assay worked, but ≥4 assays were successful.

WT, wild type.

### pTEN protein expression

#### Concordance between laboratories for corresponding whole sections

Each of the 97 whole sections was stained with pTEN and the results were compared between the two laboratories. In all pTEN-positive cases, tumour cells appeared to show cytoplasmic distribution of the staining, with a variable proportion showing in addition, some nuclear staining. There were 88 (90.7%) concordant cases, 6 (6.2%) discordant cases and 3 (3.1%) cases where data were only available from one lab. For these latter three tumours, the majority of the tissue had become detached from the slide during the antigen retrieval stage, leaving insufficient material to score. The six discordant cases were discussed and reviewed jointly by both laboratories using virtual slide conferencing (http://www.virtualpathology.leeds.ac.uk) resulting in eventual agreement in all cases (table 3).

**Table 3:** pTEN discrepant cases between the two laboratories

pTEN discrepant case	Leeds result	Cardiff result	Consensus result
3018	Positive	Negative	Positive
26018	Positive	Negative	Positive
46031	Positive	Negative	Positive
48002	Negative	Positive	Positive
L403	Negative	Positive	Negative
V007	Positive	Negative	Positive

Discrepant cases were so labelled, where a result of the whole section staining for a particular case was generated in both laboratories, but these results differed. There were only six samples, where this was the case for pTEN.

#### Concordance between TMAs and whole section IHC

According to the design of the FOCUS4 trial, it is planned to carry out an initial screen of the IHC-based biomarkers on TMAs, to identify those tumours which are positive for pTEN, and thus require no further investigation. The corresponding whole sections from tumours which appear negative, or give an equivocal result on the TMAs, will subsequently be stained for pTEN. The two validation TMAs were therefore stained with the pTEN antibody, and the results were compared with those of the corresponding 97 whole sections. In Leeds, there were 80 concordant cases and two discordant cases. It was not possible to compare the remaining 15 tumours because 11 of them were completely missing from the TMA, due to loss from core drop-off and the cores from the remaining four cases contained no tumour cells. In Cardiff, there were 83 concordant cases and six discordant cases. The remaining eight tumours were not comparable because the cores had fallen from the TMA in seven of these, and the final tumour was not assessable on the whole section, due to insufficient tumour tissue.

#### Comparison of whole section and matched diagnostic biopsy

**Figure 4:**
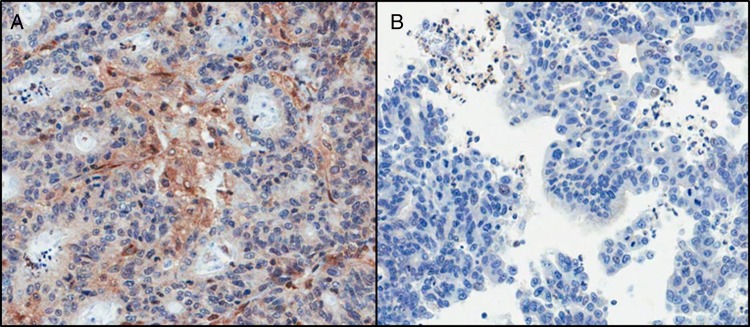
pTEN protein expression in (A) resection and (B) matched biopsy specimen, highlighting the difference in expression between both samples. The resection sample was graded as ‘no loss’ of expression, whereas the biopsy sample was graded as ‘loss of expression’ (×200 magnification).

Fourteen resection samples also had the matched biopsy available for a pTEN protein expression comparison. Only 8/14 (57.1%) gave concordant results, with both the biopsy and the resection being positive. In four samples, the biopsy was scored negative, whereas the resection was positive (figure 4). One case showed the reverse of this, while in the final case, the resection was positive, but the biopsy was equivocal, and hence unscorable.

### MMR IHC

An initial screen was carried out on the two validation TMAs for all four MMR antibodies. As with pTEN, it was planned that any tumours appearing negative or equivocal, or having insufficient material on the TMA for analysis, would have the corresponding whole section stained.

The results are given in table 4. In terms of the numbers of tumours giving a discrepant result (ie, where there was a result submitted by each lab, and this result differed), there were only three discrepant cases, although for one tumour (V007) the result for each of the MMR proteins differed (table 5). Each case was reviewed jointly, with the two laboratories using virtual slide conferencing (http://www.virtualpathology.leeds.ac.uk) and a consensus score was agreed upon for each case.

**Table 4:** Summary of mismatch repair (MMR) immunohistochemistry, showing the distribution of positive and negative cases for each antibody in both labs

Laboratory	MMR marker	Positive cases (%)	Negative cases (%)	No result or equivocal (%)
Leeds	MLH1	90 (92.8)	6 (6.2)	1 (1.0)
Cardiff	MLH1	90 (92.8)	6 (6.2)	1 (1.0)
Leeds	MSH2	95 (97.9)	1 (1.0)	1 (1.0)
Cardiff	MSH2	96 (99.0)	1 (1.0)	0
Leeds	MSH6	92 (94.8)	3 (3.1)	2 (2.1)
Cardiff	MSH6	94 (96.9)	3 (3.1)	0
Leeds	PMS2	88 (90.7)	7 (7.2)	2 (2.1)
Cardiff	PMS2	89 (91.8)	7 (7.2)	1 (1.0)

For each protein, the same number of negative cases was identified in both labs.

**Table 5:** Mismatch repair (MMR) discrepant cases between the two laboratories

MMR marker	Discrepant case(s)	Leeds result	Cardiff result	Consensus result
MLH1	V007	Positive	Negative	Positive
MSH2	V007	Positive	Negative	Positive
MSH6	V007	Positive	Negative	Positive
PMS2	V007	Positive	Negative	Positive
PMS2	V442	Negative	Positive	Negative

Discrepant cases were so labelled, where a result of the whole section staining for a particular case was generated in both laboratories, but these results differed. One sample (V007) was discrepant for all four proteins, and one other case (V442) gave a discrepant result for PMS2 only.

## Discussion

The FOCUS4 clinical trial is designed to provide a further significant step forward on the road to more effective biomarker driven, adaptive trial design in solid tumour oncology, where the opportunity to add putative predictive biomarkers to the current panel will be possible. In order for this type of trial to succeed, there must be complete confidence in the abilities of the laboratories carrying out the biomarker assay procedures. To this end, the rigorous pre-trial analytical validation was meticulously planned and carried out over several months in both laboratories, with 97 anonymised advanced CRC FFPE resection blocks and 14 matched biopsy blocks. It needed to be demonstrated that the samples could be processed and concordant results could be obtained in a timely manner. By transferring the samples between laboratories for processing, this ensured that each site was independently responsible for the total assay procedure from preparation of their own sections to the completion of assay, including interpretation, scoring and reporting of the results, and in transferring these directly to the MRC for collation; this kept each laboratory blinded to the results obtained in the other laboratory.

There was a very high concordance rate between the pyrosequencing results. Out of 97 resection samples assayed across all mutation hotspots, there were only two samples producing a different outcome, both within *PIK3CA*. It has to be acknowledged that the sensitivity of pyrosequencing lies somewhere between 5% and 25% mutant alleles in a background of WT alleles, so that in cases where low-level mutations are present, they may not always be detected. Tumour heterogeneity is a further complicating factor which could account for the failure to detect a mutation, particularly where a different part of the same FFPE block is sampled for testing^13^ as was the case in this validation exercise. It is acknowledged that the in-house pyrosequencing assays have slight variations in the primer and probe sequences. The high concordance rates during this validation, and a previous validation prior to the FOCUS3 trial,^12^ have convinced us that these differences are non-consequential. During the course of the FOCUS4 trial, it is expected that both laboratories will advance to a Next Generation Sequencing platform, revalidate the changed technique and in doing so, increase the sensitivity of mutation detection to between 1% and 5%.

The fact that there were only six (6/97, 6.2%) cases discordant between the two laboratories for pTEN, and only five (5/388, 1.3%) discrepancies for the MMR antibodies, was reassuring. Several factors were thought to contribute to the differences observed. These included the amount of necrosis observed within the tumour, and also the presence of excessive levels of mucin. Staining artefacts such as over-counterstaining and ‘edge effect’ were also noted to cause interpretation difficulties. One tumour (V007), which showed discrepant results for each of the five antibodies, was a signet-ring cell cancer. A large proportion of signet-ring cells were found to be bloated with mucin and as a result, had very scant cytoplasm available to show the presence of pTEN staining in a 5-µm thick section. A consensus opinion was agreed for each protein, but it was felt that in future, tumours showing unusual morphology should undergo joint review by both laboratories prior to reporting the results.

When the 14 matched biopsy samples were stained with the pTEN antibody, it was surprising to see that six of the tumours showed discrepancies between the biopsy and resection. In order to understand this further, an interrogative approach was adopted towards the samples and their processing, initially focused on biopsy material. It transpired that in the five samples where the biopsy was negative or equivocal and the resection specimen positive, that the biopsies had been taken and processed in one hospital, where at the time, acidified formalin-fixation was routinely used, while the resection specimen was taken in another hospital, where neutral buffered formalin was used. From the extensive data gathered for the COIN Trial,^8^ the use of acidified formalin appeared to have ceased in all laboratories in the UK since 2005 because of its deleterious effect on DNA, with formol saline or neutral buffered formalin being adopted widely as less deleterious fixatives.

Recent work has focused on the poor reproducibility of pTEN IHC scoring^14^ and indeed within the published literature the quoted rates of pTEN-negative tumours in CRC vary greatly. Here we have rigorously undertaken analysis of this biomarker, including full workup from FFPE blocks in two independent laboratories and following independent scoring, have shown closely adherent results. The addition of cell-line control TMAs to each slide will continue throughout the FOCUS4 trial.

It has been demonstrated here that two reference laboratories can independently obtain highly concordant biomarker results across a wide panel of assays on a large number of samples. Unfortunately, there was insufficient material remaining at each laboratory to repeat all assays and ascertain the local reproducibility rates for all assays. This exercise has highlighted issues which can potentially make interpretation more difficult, so that should they arise during the course of the FOCUS4 trial, a web-linked protocol is now in place to allow joint discussion between laboratories. It has also been agreed that a continuous inter-laboratory validation will take place for the duration of the trial, with each laboratory supplying three samples on alternate months to the other laboratory for confirmatory testing. Each laboratory will also continue to participate in the UK NEQAS molecular genetic analysis of CRC external quality assurance (EQA) scheme; this currently (as of 2013) includes analyses for *KRAS, NRAS*, *BRAF* and *PIK3CA*. With all these measures in place, we are confident that molecular testing will continue to be delivered with high and exacting standards from these designated testing laboratories. Going forward in personalised medicine trials, we would recommend the use of centralised testing, identical protocols, pre-trial validation of techniques, continuous quality control and independent review of the testing by the trials unit.

**Take home messages**

- With many clinical trials reliant upon biomarker analyses for patient randomisation, it is imperative that confidence in the laboratories carrying out the analyses is established. The inter-laboratory validation carried out here is an example of how this can be established.
- Of equal importance is the identification of issues which make the interpretation of biomarker results difficult, and the putting into place mechanisms to overcome this.
- Laboratories must be transparent regarding their assay validations, in order to inspire the necessary levels of confidence in their abilities. Publication would seem to be the obvious route to take to ensure this.

## Supplementary Material

Web supplement
