# Supplementary material for: Pre-trial inter-laboratory analytical validation of the FOCUS4 personalised therapy trial
Source: J Clin Pathol. 2015 Sep 8;69(1):35–41. doi: 10.1136/jclinpath-2015-203097 (PMC4717430; doi:10.1136/jclinpath-2015-203097)
Supplement: Web supplement [file jclinpath-2015-203097-s1.pdf]

APPENDIX 1

Codon	Primer	Primer sequence
KRAS12/13	PCR-F	GGCCTGCTGAAAATGACTGA
	PCR-R*	AGCTGTATCGTCAAGGCACTCT
	Sequencing	AAACTTGTGGTAGTTGGA
KRAS61	PCR-F	AATTGATGGAGAAACCTGTCTCTT
	PCR-R*	TCCTCATGTACTGGTCCCTCATT
	Sequencing	GGATATTCTCGACACAGC
KRAS146	PCR-F*	TCAGGACTTAGCAAGAAGTTATGG
	PCR-R	TGCAGAAAACAGATCTGTATTTAT
	Sequencing	GTGTTACTTACCTGTCTTGT
BRAF	PCR-F	TGAAGACCTCACAGTAAAAATAGG
	PCR-R*	TCCAGACAACTGTTCAAACTGAT
	Sequencing	TGATTTTGGTCTAGCTACA
NRAS12/13	PCR-F	CTTGCTGGTGTGAAATGACTGAG
	PCR-R*	TGGATTGTCAGTGCGCTTTTC
	Sequencing	CTGGTGGTGGTTGGA
NRAS61	PCR-F*	GAAACCTGTTTGTTGGACATACTG
	PCR-R	TCGCCTGTCCTCATGTATTG
	Sequencing	CTCTCATGGCACTGTACT
PIK542	PCR-F*	AAAGCAATTTCTACACGAGATCC
	PCR-R	GCACTTACCTGTGACTCCATAGA
	Sequencing	TTCTCCTGCTCAGTGAT
PIK545-6	PCR-F	ACAGCTCAAAGCAATTTCTACACG
	PCR-R*	TCCATTTTAGCACTTACCTGTGAC
	Sequencing	GATCCTCTCTCTGAAATC
PIK1047	PCR-F*	TGAGCAAGAGGCTTTGGAGTAT
	PCR-R	TGCTGTTTAATTGTGTGGAAGATC
	Sequencing	GTTGTCCAGCCACCA

A: Pyrosequencing primer sequences used in the Leeds laboratory. PCR-F, forward PCR primer sequence; PCR-R, reverse PCR primer sequence; *indicates the biotinylated primer.

Codon	Primer	Primer sequence
KRAS12/13	PCR-F	GACTGAATATAAACTTGTGGTAGTTGG
	PCR-R*	TTGGATCATATTCGTCCACAA
	Sequencing	CTTGTGGTAGTTGGAG
	Sequencing[1]	TGTGGTAGTTGGAGCTG
KRAS61	PCR-F	CAGGAAGCAAGTAGTAATTGATGG
	PCR-R*	AAGAAAGCCCTCCCCAGTC
	Sequencing	GGATATTCTCGACACAGC
KRAS146	PCR-F	AGGCTCAGGACTTAGCAAGAAGTT
	PCR-R*	GATTAAGAAGCAATGCCCTCTC
	Sequencing	GGAATTCCTTTTATTGAAAC
BRAF	PCR-F	AAGACCTCACRGTAAAAATAGGTG
	PCR-R*	CCAGACAACTGTTCAAACTGAT
	Sequencing	ATAGGTGAYTTTGGTCTAG
NRAS12/13	PCR-F	GGTTTCCAACAGGTTCTTGCTGGTG
	PCR-R*	ACAGGATCAGGTCAGCGGGCT
	Sequencing	TGGTGGTGGTTGGAG
	Sequencing[2]-P2	TGGTGGTGGTTGGAGCA
	Sequencing[3]-P3	TGGTGGTTGGAGCAGG
NRAS61	PCR-F*	TCCACACCCCCAGGATTCTT
	PCR-R	CGCCTGTCCTCATGTATTGGTCT
	Sequencing	CTCATGGCACTGTACTCT
PIK542-6	PCR-F	GCACTTACYTGTGACTCCATAGAA
	PCR-R*	GAAAATGACRAAGAACAGCTCAAA
	Sequencing	CCTGTGACTCCATAGAAAA
PIK1047	PCR-F	GTTCAATGCATGCTGTTTAATTGT
	PCR-R*	CGAAAGACYCTAGCCTTAGATAAA
	Sequencing	TTTGTTGTCCAGCCA

B: Pyrosequencing primer sequences used in the Cardiff laboratory. PCR-F, forward PCR primer sequence; PCR-R, reverse PCR primer sequence; *indicates the biotinylated primer; [1]used to distinguish between c.35G>T or c.37G>T; [2,3]used for characterisation of unusual mutations (P refers to position of mutation).
